# PROTOCOL: Effectiveness of interventions for people with disabilities in low‐ and middle‐income countries—an evidence and gap map

**DOI:** 10.1002/cl2.1006

**Published:** 2019-07-05

**Authors:** Ashrita Saran, Howard White, Hannah Kuper

**Affiliations:** ^1^ Campbell Collaboration Delhi India; ^2^ Director of the International Centre for Evidence in Disability London School of Hygiene and Tropical Medicine (LSHTM) London UK

## 1 BACKGROUND

### 1.1 The problem, condition or issue

Disability is an umbrella term, covering impairments, activity limitations and participation restrictions. The Preamble to the United Nation Convention on the Rights of Persons with Disability (UNCRPD) acknowledges that disability is “an evolving concept,” but also stresses that “disability results from the interaction between persons with impairments and attitudinal and environmental barriers that hinder their full and effective participation in society on an equal basis with others.” An impairment becomes disabling when individuals are prevented from participating fully in society because of social, political, economic, environmental, or cultural factors.

More than one billion persons in the world have some form of disability. This corresponds to about 15% of the world's population (World Health Organisation [WHO, 2011]). The majority of people with disabilities (80%) live in low‐ and middle‐income countries (LMICs), and disability is believed to affect disproportionately the most disadvantaged sector of the population (Banks, Kuper, & Polack, 2017). People with disabilities are more likely to experience a range of exclusions, including from employment, education, health care access and social participation (WHO, 2011). As a consequence, people with disabilities are more likely to experience poverty because disability causes poverty, but also because people who are poor are more likely to become disabled (WHO, 2011). The impact of disability on poverty is also borne at a global level (Banks et al., 2017). In 2004, the World Bank estimated the global GDP loss due to disability to be between $1.71 trillion and $2.23 trillion annually (Metts & Mondiale, 2004); between 12% and 20% of the populations of developing countries were thought to be nonproductive due to disability (Mondiale, 2007).

A key argument in attaining welfare for people with disabilities is to equalise social and economic opportunities from both humanitarian and economic perspectives. From a humanitarian perspective, it is to secure basic human rights for people with disabilities. From an economic perspective, it is expected to increase the human capital of people with disabilities, and thus enable them to reduce their dependence on income transfers and other forms of public support. This economic expectation addresses disability as a development issue. Research is now required to determine the most cost‐effective ways to overcome the above obstacles and develop disability policies and strategies that increase the economic contributions of people (Metts & Mondiale, 2004).

In recognition of this point, disability is referenced in various parts of the sustainable development goals (SDGs) (United Nations—Disability Department of Economic and Social Affairs) related to education, growth and employment, inequality and accessibility of human settlements. Furthermore, SDG 17 stresses that in order to strengthen the means of implementation and revitalise the global partnership for sustainable development, the collection of data, monitoring and accountability of the SDGs are crucial. Significantly increasing the availability of high‐quality, timely and reliable data that is also disaggregated by disability is one of the key mandates. Evidence and gap maps (EGMs) can contribute to achieving SDG 17 by supporting the prioritisation of global evidence synthesis needs and primary data collection.

Disability is also a human rights issue, and this is highlighted in a range of international documents, including the World Programme of Action Concerning Disabled People (WPA, 1982), the Convention on the Rights of the Child (CRC, 1989), the Standard Rules on the Equalisation of Opportunities for People with Disabilities (1993), and most importantly the United Nations Convention on the Rights of Persons with Disabilities (UNCRPD, 2006). The UNCRPD aims to “promote, protect and ensure the full and equal enjoyment of all human rights and fundamental freedoms by all persons with disabilities, and to promote respect for their inherent dignity.” It reflects the major shift in global understanding and responses towards disability, and emphasises that people with disabilities have the right for full inclusion.

Inclusive development is that which includes and involves everyone, especially those who are marginalised and often discriminated against (United Nations Development Programme, 2010). Unless people with disabilities are brought into mainstream it is impossible to cut the cycle of poverty and discrimination. Attention to disability issues is now increasingly being seen in the policies and programmes of bilateral agencies like Department of International Development (DFID, 2000) either as part of inclusive new policies or in disability‐specific initiatives, many of which are linked either implicitly or explicitly to poverty alleviation efforts or public health initiatives as United States Agency for International Development (USAID, 1997). Although there is little data on the cost‐effectiveness of disability‐inclusive development, the Asian Development Bank (ADB) maintains that the costs associated with including people with disabilities are far outweighed by the long‐term financial benefits to individuals, families and society (ADB, 2005).

To enable people with disabilities to contribute to creating opportunities, share in the benefits of development, and participate in decision‐making, a twin‐track approach may be required (DFID, 2000). The “Twin‐Track approach” aims to break this cycle between disability, poverty and exclusion, by both empowerment of individuals/families/organisations and by breaking down barriers in society, and is advocated for by many international donors (e.g., the World Bank, DFID, the German Cooperation; the European Community [EC] and the Finnish Cooperation) and non‐governmental organisations (NGOs). The Twin‐track approach promotes integration of disability‐sensitive measures into the design, implementation, monitoring and evaluation of all development policies and programmes, called as “mainstreaming disability,” while simultaneously undertaking “targeted measures” such as disability‐specific policies, programmes and initiatives to ensure the inclusion and full enjoyment of human rights by persons with disabilities (United Nations Development Programme, 2010).

The WHO community‐based rehabilitation (CBR) guidelines is based on this approach. CBR is a multisectoral, bottom‐up strategy which can ensure that the Convention on Rights of People with Disabilities (ILO/UNESCO/WHO, 2004) makes a difference at the community level. While the UNCRPD provides the philosophy and policy, CBR is a practical strategy for implementation of disability‐inclusive development (Helander, 1989). CBR activities are designed to meet the basic needs of people with disabilities, reduce poverty, and enable access to health, education, livelihood and social opportunities—all these activities fulfil the aims of the UNCRPD.

Guidelines to generate an inclusive and global dialogue, implementing the SDGs must be in line with and build upon existing international and national commitments and mechanisms. The WHO's CBR recognises CBR as a comprehensive and multisectoral strategy to equalise opportunities and include people with disabilities in all aspects of community life. Therefore, the CBR will serve as a guiding framework and the five pillars of CBR: health, education, livelihood, social and empowerment will form the intervention and outcome categories.

### 1.2 Why is it important to do the EGM?

Over the past decade the academic literature on disability outcomes and effectiveness has grown substantially (Andresen, Lollar, & Meyers, 2000; Devon, Lydon, Healy, & McCoy, 2016; Iemmi et al., 2015). Several important questions have not been adequately addressed, however. For example, what type of evidence is needed, and what are realistic expectations for disability outcomes and effectiveness research? A lack of rigorous and comparable data on disability and evidence on programmes that work can impede understanding and action. Understanding the numbers of people with disabilities and their circumstances can improve efforts to remove disabling barriers and provide services to allow people with disabilities to participate on an equal basis with others. For example, better measures of the environment and its impacts on the different aspects of disability need to be developed to facilitate the identification of cost‐effective environmental interventions.

Knowledge production takes place across several sectors (health, social welfare and education), focuses on various populations (different ages, ethnicities, or with different needs), and involves rather diverse methodical approaches (e.g., systematic reviews, primary studies of different designs, etc.). A mapping of the existing knowledge base is therefore required to provide a comprehensive overview of existing knowledge in this area and enable the purposeful and targeted commissioning of future research, tailored to the most eminent needs for knowledge and guidance. This ambition could be fulfilled by proposed EGM.

## 2 OBJECTIVES

The proposed EGM will present studies of the effectiveness of these interventions across a range of outcome domains. Specifically, the objectives of the map are to

1. Develop a clear framework of types of interventions and outcomes related to effectiveness of interventions for people with disabilities in LMICs.
2. Map available systematic reviews and primary studies on the effectiveness of disability interventions in low‐ and middle‐income countries in this framework, with an overview provided in a summary report.
3. Provide database entries of included studies which summarise the intervention, context, study design and main findings.

## 3 METHODOLOGY

EGMs provide a visual overview of the availability of evidence for a particular sector—in this case will include “people with disabilities.” The EGM will consolidate what we know and do not know about “what works” by mapping out existing and ongoing systematic reviews and impact evaluations in this field; and by providing a graphical display of areas with strong, weak or nonexistent evidence on the effect of interventions or initiatives.

The EGMs are presented in two dimensions: the rows list interventions and the column list outcome domains. Each cell shows studies which contain evidence on that combination of intervention and outcomes. This EGM will provide an overview of the existing systematic reviews and impact evaluations on the key outcome domains and interventions aimed to increase the welfare of people with disabilities in LMICs.

This EGM will be populated based on the following criteria (Appendix A):

- Criteria for including and excluding studies
- Types of studies to be included
- Quality ratings using Assessing Methodological Quality of Systematic Reviews (AMSTAR‐2).

### 3.1 Types of study designs

The EGM will include systematic reviews of effects of interventions and effectiveness studies that used either (a) randomised experimental design, (b) rigorous quasi‐experimental design, (c) natural experiments, (d) regression discontinuity, (e) propensity score matching, (f) difference in difference, (g) instrumental variables, (h) other matching designs and (i) single‐subject designs.

### 3.2 Status of studies

EGM will include both completed and on‐going studies. Ongoing studies which are in‐progress or the full review is not yet published. Usually for such studies protocols might have been published.

### 3.3 Population

The target populations are people with disabilities living in LMICs based on World Bank Classifications (2016). People with disabilities include those who have long‐term physical, mental, intellectual, or sensory impairments, which in interaction with various barriers may hinder their full and effective participation in society on an equal basis with others (Iemmi et al., 2015).

In recent years, the inclusion of traditionally underrepresented groups in research has received increasing attention, including racial and ethnic minorities, women, elderly individuals and children (Glickman et al., 2008). Also, some of the population groups are more affected by the outcomes of disability. The 2010 MDG report is the first to mention disabilities, noting the limited opportunities facing children with disabilities, and the link between disability and marginalisation in education. Similarly, the disability prevalence among people 45 years and older in low‐income countries is higher than in high‐income countries, and higher among women than among men (Üstün, Murray, & Evans, 2003).

Hence, the population subgroups of interest for this EGM include: women, vulnerable children (particularly children in care), conflict (conflict and postconflict settings), migrants and ethnic minority groups.

Studies with multiple populations are included in the map as long as they have a LMIC focus. For reviews with global focus, we will include them as eligible if they did not have any search restriction.

### 3.4 EGM framework outcomes

The five main outcome categories are as mentioned below and they are plotted against the WHO's CBR indicators (Table 1)

1. Health
2. Education
3. Livelihood
4. Social
5. Empowerment.

**Table 1:** Outcome categories and subcategories

Outcome	WHO's community‐based rehabilitation (CBR) indicators
*Health component*	
Mental health and cognitive development	Men, women, boys and girls with disability equally access mental health services and engage in activities needed to achieve the highest attainable standard of mental health services
Access to health services	Men, women, boys and girls with disability equally access health services and engage in activities needed to achieve the highest attainable standard of health
	Percentage of people with disabilities and their families that have access to medical care
	Men, women, boys and girls with disability feel they are respected and treated with dignity when receiving health services
Immunisation	Percentage of people with disabilities who receive full immunisation as recommended for their country by WHO
Health check‐up	Men, women, boys and girls with disability know how to achieve good levels of health and participate in activities contributing to their health
	Percentage of children with disability who receive the recommended health check‐ups
Rehabilitation services	Men, women, boys and girls with disability engage in planning and carry out rehabilitation activities with the required services
Access to assistive devices	Men, women, boys and girls with disability have access to, use, and know how to maintain appropriate assistive products in their daily life
*Nutrition*	
Morbidity and mortality	Men, women, boys and girls with disability access and benefit from quality medical services appropriate to their life stage needs and priorities
*Education*	
Enrolment to primary, secondary, and tertiary education	Policies and resources are conducive to education for people with disabilities and ensure smooth transitions through different stages of learning
	Children with disability participate in and complete quality primary education in an enabling and supportive environment
	Men, women, boys and girls with disability have resources and support to enrol and complete quality secondary and higher education in an enabling and supportive environment
	Youth with disability experience post school options on an equal basis with their peers
Attendance	Men, women, boys and girls with disability have resources and support to enrol and complete quality secondary and higher education in an enabling and supportive environment
Education in mainstream education facilities/inclusive education	Percentage of people with disabilities who acquire education in mainstream education facilities
Social and life skill development	Men, women, boys and girls with disability make use of youth or adult centred learning opportunities to improve their life skills and living conditions
Learning and achievement	Men, women, boys and girls with disability experience equal opportunities to participate in learning opportunities that meet their needs and respect their rights
Access to educational services	Children and youth with disability participate in a variety of nonformal learning opportunities based on their needs and desires
	Children with disability actively participate in early childhood developmental activities and play, either in a formal or informal environment
*Livelihood*	
Employment in formal and informal sector	Men and women with disability have paid and decent work in the formal and informal sector on equal bases with others
	Women and men with disability earn income through their own chosen economic activities
	Youth and adults with disability acquire marketable skills on an equal basis with others through a range of inclusive training opportunities
Access to job market	
Control over own money	Women and men have control over the money they earn
Access to financial services such as grants and loans	Men and women with disability have access to grants, loans and other financial services on an equal basis with others
	Men and women with disability participate in local saving and credit schemes
Poverty and out‐of‐pocket payment	Percentage of people with disabilities who are covered by social protection programmes
Access to social protection programmes	Men and women with disability access formal and informal social protection measures they need
Participation in development of inclusive policies	Inclusive policies, practices and appropriate resources, defined with people with disabilities enable equal participation of women and men with disability in livelihood (training, finance, work opportunities, and social protection)
*Social*	
Stigma and discrimination	Communities have increased awareness about disability, with a reduction in stigma and discrimination towards people with disabilities
Safety	Men, women, boys and girls with disability feel safe in their family and community
Participation in mainstream recreational, leisure and sports activity	Men, women, boys and girls with disability participate in inclusive or specific recreation, leisure and sports activities
Legal rights	All people with disabilities (PwD) are recognised as equal citizens with legal capacity
Access to justice	PwD access and use formal and informal mechanisms of justice
Participation in cultural and religious activity	Men, women, boys and girls with disability participate in artistic, cultural or religious events in and outside their home as they choose
Interpersonal interaction and relationships	Men, women, boys and girls with disability experience support of the community and their families to socialise and form age‐appropriate and respectful relationships
	Percentage of people with disabilities who feel respected in their decisions regarding personal relationships
Social identity and responsibilities	Men, women, boys and girls with disability feel valued as community members and have a variety of social identities, roles, and responsibilities
*Empowerment*	
Informed choices	PwD make informed choices and decisions
Positions in public institutions and Judiciary	Men and women with disability participate in political processes on an equal basis with others
Voting rights	Men and women with disability participate in political processes on an equal basis with others
Representation at community level	PwD actively engage in and benefit from self‐help groups in the local communities, if they choose (inclusive or specific)
	Self‐help groups come together to form federations to harness collective energy and influence positive change
	Men and women with different kinds of disability living in different situations (rural or urban areas, poor or rich, refugees) feel they are adequately represented by DPO
Advocacy	Men, women, boys, and girls with disability effectively use communication skills and resources (including supportive decision‐making) to facilitate interactions and influence change
	Men, women, boys, and girls with disability play a catalysering role in mobilising key community stakeholders to create an enabling environment

### 3.5 Types of interventions

As indicated in SDG guidelines to generate an inclusive and global dialogue, implementing the SDGs must be in line with and build upon existing international and national commitments and mechanisms. The WHO's CBR recognises CBR as a comprehensive and multisectoral strategy to equalise opportunities and include people with disabilities in all aspects of community life. Therefore, the CBR will serve as a guiding framework for the intervention and outcome categories as listed below in order to realise the full inclusion and empowerment of persons with disabilities. We have added “Advocacy and Governance” as one of the components as strong advocacy may be required to prevent and/or address abuse, neglect and exploitation that people with intellectual and/or developmental disabilities may experience (CBM, 2012). People with disabilities may need the support of advocates to become effective self‐advocates.

The included interventions cover all main strategies to reduce disability related outcome. The six main intervention categories are

1. Health
2. Education
3. Livelihood
4. Social
5. Empowerment
6. Advocacy and Governance.

Table 2 lists the intervention subcategories under each of these headings.

**Table 2:** Intervention subcategories

CBR Pillar (intervention category)	Component (intervention subcategory)	Examples
Health	Promotion	Parent/family training and education, support health promotion campaigns and health care provider training
	Prevention	Avoidance of war; improvement of the educational, economic and social status of the least privileged groups; identification of types of impairment and their causes within defined geographical areas; introduction of specific intervention measures through better nutritional practices; improvement of health services, early detection and diagnosis; prenatal and postnatal care; proper health care instruction, including patient and physician education; family planning; legislation and regulations; modification of life‐styles; selective placement services, education regarding environmental hazards; and the fostering of better informed and strengthened families and communities
	Medical care	Periodic health screening, evaluation of traumatic injuries, access to early treatment
	Rehabilitation	Training in self‐care activities, including mobility, communication and daily living skills, with special provisions as needed, for example, for the hearing impaired, the visually impaired and the mentally retarded, vocational rehabilitation services (including vocational guidance), vocational training, cognitive behaviour therapy, cognitive stimulation, rehabilitation and training, activity therapy centres, supportive therapy, stress‐management interventions/psychosocial support, trauma informed therapy, acceptance and commitment therapy, interpersonal therapy, modification of environment, trauma informed therapies.
	Assistive devices	Provision of appliances (ortheses, prostheses, hearing aids, etc.), devices such as day calendars with symbol pictures for people with cognitive impairment, communication boards and speech synthesisers for people with speech impairment
Education	Early child development	Speech and language therapist, physiotherapy, gait training, occupational therapy
		Inclusive social services and child protection
	Nonformal	Community‐based‐sports programme, faith‐based schools, home‐based learning, play groups
		Inclusive early childhood education
	Primary	Provision of learning material and special equipment (Braille, audio cassettes, sign language, etc.)
	Secondary and higher	Recruitment and training of specialised teachers
		Resource rooms
		Bypass intervention
	Life‐long learning	Explicit social skills interventions, adult literacy programmes, continuing education, life and survival skills
Livelihood	Skills development	Training opportunities for jobs, home‐based trainings, vocational training, training in mainstream institutions and community‐based trainings
	Self‐employment	Income generation programme
	Waged employment	Realistic quota legislation in jobs and participation in labour intensive public works programmes
	Financial services	Access to credit, health insurance coverage
	Social protection	International legislation like universal declaration of human rights, Social insurance schemes, birth registration, social assistance intervention, referral services
Social	Relationship, marriage and family	Family planning accessible to disabled, media campaigns and religious leaders
	Personal assistance	Accommodation support, home modifications, self‐help groups and Disabled People Organisations (DPOs)
	Culture, religion and arts	Promoting use of art for social change like positive portrayal, silent theatres, complementary therapy in the form of art, and music. Inclusive art education, diversity trainings, encouraging inclusion in mainstream cultural programmes, work with spiritual and religious leaders, and groups
	Sports, recreation and leisure	Provision of adapted sports equipment, organisation of inclusive sports events, linking people with disabilities to mainstream recreation and sporting clubs/associations, positive media coverage of disability recreation, using recreation and sport to raise awareness about inclusion, advocate alongside disabled people's organisations and appropriate training
	Access to justice	Legal awareness, identification of available resources like local leaders, DPO's, legal centres, legal aid. Promoting legal rights and empowerment, inheritance right, community, or legal aid centre
Empowerment	Social mobilisation	Find about the community
		Building trust and credibility within community
		Raise awareness in the community
		Motivate the community to participate
		Bringing stakeholder together
		Capacity building
		Celebrating achievements
	Political participation	Reservation of position in public and political institution
		Development of political awareness
		Access to political process
		Disability awareness within political system
	Language and communication	Speech and language therapy, deaf clubs, stroke clubs, self‐advocacy, interventions removing communication barriers
	Self‐help groups and Disabled People's Organisations	Creating joint resources like training material, community directories, advocating rights of persons with disability, partnership with existing self‐help groups
Advocacy and Governance		National prevention programmes against certain illnesses (polio, leprosy)
		Establishment/reinforcement of a Special Education Service in the Ministry of Education
		Establishment/reinforcement of medical rehabilitation centres
		Legislative reforms: elimination of all forms of discrimination
		Mandating healthy behaviour as childhood immunisation/seat belts etc.
		Raising awareness on human rights through media
		Appropriate budgetary allocation

### 3.6 Systematic reviews

The search will be conducted in three stages

1. Populating the map based on a search of systematic reviews
2. Populating the map based on search of primary studies
3. Populating the map based on grey literature search.

Search will be as comprehensive as possible, using (but not limited to) relevant systematic review database for first stage along with bibliographic databases (Appendix B), EGM databases, web‐based search engines, websites of specialist organisations, bibliographies of relevant reviews, and targeted calls for evidence using professional networks or public calls for submission of articles. Database for EGMs will also be searched to identify any map and relevant populated studies. Additionally, reference lists of the included reviews will be reviewed and the authors contacted for information on other relevant sources. Citation searches will be performed and databases like Web of Science, Scopus and Google Scholar will be searched (Appendix C).

To identify unpublished reviews studies, we will search the following databases: Dissertation Abstracts, Conference Proceedings and Open Grey.

To identify ongoing studies, we will search ClinicalTrials.gov and WHO International Clinical Trials Registry Platform and CENTRAL Trials Register within the Cochrane Library will be used for published trials.

We will assess the methodological quality of each included systematic review using AMSTAR‐2 (Shea et al., 2007). The assessments will be carried out by two reviewers independently.

## 4 DIMENSIONS

The EGM will have two primary dimensions: interventions (rows) and outcomes (columns). Additional dimensions will be

1. Population subgroups of interest include: age group (under five, children, adolescent and elderly), women, vulnerable children (particularly children in care), conflict (conflict and postconflict settings), migrants and ethnic minority groups
2. Study designs
3. Region
4. Country.

In the hard copy of the EGM, multiple 2 × 2 representations of the EGM will be reported. A copy of the coding form will be included as an annex to the EGM report.

In the online version, the additional dimensions will be possible to use as a filter. The online version will include references to included studies and brief summaries of each study based on the abstract (for primary studies) or plain language summary (for systematic reviews) provided for it. Primary studies included in systematic reviews will be highlighted.

In the EGM report, we will

- summarise the findings of the EGM
- present areas of particular interest in depth (e.g., areas of strong evidence; substantial evidence gaps; the prevalence of evidence by geographical region; the prevalence of evidence by gender or service setting etc.)
- present potential implications for policy, practice and research
- provide a plain language statement of the EGM findings.

## 5 CODING/CLASSIFICATION

We will code each included study using a piloted coding tool covering study characteristics, population, intervention and outcomes (Appendix D Coding tool).

## 6 STAKEHOLDER ENGAGEMENT

An advisory group consisting of international experts in disability will contribute to the preparation of the EGM by commenting on protocol drafts. Suggested members for this advisory panel are

- Dr Tom Shakespeare
  He is Professor of Disability Research, Norwich Medical School. His primary research interests are in disability studies, medical sociology, and in social and ethical aspects of genetics. He has had a long involvement with the disabled people's movement in United Kingdom and internationally. In the context of disability arts, he has also been active in arts and culture, and was a member of Arts Council England from 2003 to 2008. During his 5 years at WHO, he helped produce and launch key reports such as the world report on disability (WHO, 2011) and International Perspectives on Spinal Cord Injury (WHO, 2013), and was responsible for the UN statement on forced, coerced, and otherwise involuntary sterilisation (WHO, 2014).
- Dr David Olichini: He is the head of Prevention and Health Unit, NCDs Technical Advisor, Handicap International Federation.

## 7 ROLES AND RESPONSIBILITIES

- *Content expertise*:

Dr Hannah Kuper, Director of the International Centre for Evidence in Disability, a research group at LSHTM that works to expand the research and teaching activities of LSHTM in the field of global disability. Her main research interest is disability in low and middle income countries, with a particular focus on assessment of the prevalence of disability and impairments, including in children, and development of new methods in undertaking these surveys (e.g., use of mobile technologies), investigation of the health and rehabilitation needs of people with disabilities, and how these can be met in low resources settings and research on the relationship between poverty and disability, and the potential role of social protection in breaking this cycle. She has an undergraduate degree from Oxford University in Human Sciences and a doctorate from Harvard University in epidemiology. She has worked at LSHTM since 2002.

### 7.1 Systematic review method expertise

All authors are experienced systematic reviewers, which means they are proficient in carrying out the various processes in an EGM, such as eligibility screening, quality assessment and coding.

- EGM methods expertise:
  All team members have previous experience in systematic review methodology, including search, data collection, statistical analysis, theory‐based synthesis, which mean they are proficient in carrying out the various processes in an EGM, such as search, eligibility screening, quality assessment and coding.
- Information retrieval expertise: All authors have previous experience in developing search strategies.

All authors have previous experience in developing search strategies.

## CONFLICT OF INTERESTS

The authors declare that there are no conflict of interests.

## PRELIMINARY TIMEFRAME

This EGM will be developed in two phases.

**Phase 1: Systematic reviews**

- 25 January 2018: Protocol and Literature search completed
- 15 February 2018: Study inclusion completed
- 28 February 2018: Quality assessment and coding completed
- 15 March 2018: Draft EGM submitted
- 31 March 2018: Final EGM submitted

**Phase 2: Primary studies**

- 25 January 2018: Protocol and Literature search completed
- 15 February 2018: Study inclusion completed
- 28 February 2018: Quality assessment and coding completed
- 15 March 2018: Draft EGM submitted
- 31 March 2018: Final EGM submitted

### Plans for updating the EGM

The lead author will be responsible for yearly updates of the EGM but this is also subject to financing being available.

## APPENDIX A

**Table A1:** Description of methods used for inclusion and exclusion of studies

Selection criteria	Inclusion	Exclusion
Publication year	After 2000	Before 2000
Publication status	Completed and on‐going	None
Study design	The EGM will include systematic reviews of effects of interventions and effectiveness studies that used either: (a) randomised experimental design, or (b) rigorous quasi‐experimental design, (c) natural experiments, (d) regression discontinuity, (e) propensity score matching, (f) difference in difference, (g) instrumental variables, (h) other matching design, and (i) single subject design	Literature reviews, non‐effectiveness studies, case studies and qualitative studies
Population	People with disability, and/or their family, their caregivers, their community living in low‐ and middle‐income countries	People with disabilities and/or their family, their caregivers, their community living in high‐income countries
	Disability is defined as impairments, activity limitations, and participation restrictions denoting the negative aspects of the interaction between an individual (with a health condition) and that individual's contextual factors (environmental and personal factors) (WHO, 2011, 2001)	
	For primary studies we will include participants from low‐ and middle‐income countries only, as this was the original commitment of CBR (Helander, 1989)	
Interventions	A CBR programme is formed by one or more activities in one or more of the five components (health, education, livelihood, social, and empowerment). List of activities for each element of the five components are presented within the CBR Guidelines under the section “Suggested activities” (WHO, 2010). The following activities are here given as examples:• Health: training PWD in the use of assistive devices; providing information to PWD and their family or their caregivers about time and location of activities for screening health conditions and impairments associated with disabilities.• Education: providing education and training for families or caregivers of PWD; installing ramps in schools to make them accessible to PWD using wheelchairs.• Livelihood: linking the jobseeker with disability to existing support services; advocating before relevant public and private agencies to ensure accessible housing for PWD.• Social: converting institutions for PWD in rehabilitation centres; providing information to PWD about the sports opportunities available within the community.• Empowerment: helping PWD running meetings of new self‐help group; involving disabled's people organisations in CBR planning, implementation, and monitoring	Interventions not focused on people with disabilities. We will also exclude studies that deals temporary or reversible form of disability for examples, maternal depression or back pain
Outcome	We will use the CBR framework for outcomes	None
Quality	We will not restrict based on quality	None

## APPENDIX B

**Table B1:** List of databases

Indexes	
*International Organizations*	
ILO	
DFID (including Research for Development (R4D)	
UNESCO	
WHO	
Disability Programme of the United Nations Economic and Social Commission for Asia and the Pacific (UNSCAP	
United States Agency for International Development (USAID)	
*Evidence and Gap Map database*	
3ie Evidence and gap map repository	
Swedish Agency For Health Technology Assessment and Assessment of Social Services	
Collaboration for Environmental Evidence	
Global Evidence Mapping Initiative	
Evidence based Synthesis Program (Department of Veteran affairs)	
Cochrane	
Evidence based policing matrix	
EPPI Centre Evaluation Database of Education Research	
*Systematic review database*	
Cochrane	
Campbell	
3ie Systematic Review Database	
Research for Development	
Epistemonikos	
*Academic databases*	
Econlit	
The National Bureau of Economic Research (NBER)	
Social Science Research Network (SSRN)	
International Bibliography of Social Sciences (IBSS)	
Applied Social Sciences Index and Abstracts (ASSIA)	
Embase	
PsycINFO	
MEDLINE	
WHO's Global Health Library	
CABI's Global Health	
ERIC	
CINHAL	
SCOPUS	
Web of Science	

## APPENDIX C

**Table C1:** Search string

Search string/key words (for Ovid Medline platform)
Developing Country Free Text
− (developing OR less‐developed OR less* developed OR ‘‘under developed’’ OR underdeveloped OR under‐developed OR middle‐income OR ‘‘middle income’’ OR ‘‘low income’’ OR low‐income OR underserved OR ‘‘under served’’ OR deprived or poor*) adj3 (countr* OR nation OR population OR world OR state OR economy OR economies).mp
− (‘‘third world’’ OR L&MIC OR L&MIC OR LAMIC OR LDC OR LIC OR LMIC* OR lami countr* OR transitional countr*).mp
− (Africa OR “Sub‐Saharan Africa” OR “North Africa” OR “West Africa” OR “East Africa” OR Algeria OR Angola OR Benin OR Botswana OR Burkina Faso OR Burundi OR Cameroon OR “Cape Verde” OR “Central African Republic” OR Chad OR “Democratic Republic of the Congo” OR “Republic of the Congo” OR Congo OR “Cote d'Ivoire” OR “Ivory Coast” OR Djibouti OR Egypt OR “Equatorial Guinea” OR Eritrea OR Ethiopia OR Gabon OR Gambia OR Ghana OR Guinea OR Guinea‐Bissau OR Kenya OR Lesotho OR Liberia OR Libya OR Madagascar OR Malawi OR Mali OR Mauritania OR Morocco OR Mozambique OR Namibia OR Niger OR Nigeria OR Rwanda OR “Sao Tome” OR Principe OR Senegal OR “Sierra Leone” OR Somalia OR Somaliland OR “South Africa” OR “South Sudan” OR Sudan OR Swaziland OR Tanzania OR Togo OR Tunisia OR Uganda OR Zambia OR Zimbabwe).mp
− (“South America” OR “Latin America” OR “Central America” OR Mexico OR Argentina OR Bolivia OR Brazil OR Chile OR Colombia OR Ecuador OR Guyana OR Paraguay OR Peru OR Suriname OR Uruguay OR Venezuela OR Belize OR “Costa Rica” OR “El Salvador” OR Guatemala OR Honduras OR Nicaragua OR Panama).mp
− (“Middle East” OR “South‐East Asia” OR “Indian Ocean Island*” OR “South Asia” OR “Central Asia” OR Caucasus OR Afghanistan OR Azerbaijan OR Bangladesh OR Bhutan OR Burma OR Cambodia OR China OR Georgia OR India OR Iran OR Iraq OR Jordan OR Kazakhstan OR Korea OR “Kyrgyz Republic” OR Kyrgyzstan OR Lao OR Laos OR Lebanon OR Macao OR Mongolia OR Myanmar OR Nepal OR Oman OR Pakistan OR Russia OR “Russian Federation” OR “Saudi Arabia” OR Bahrain OR Indonesia OR Malaysia OR Philippines OR Sri Lanka OR Syria OR “Syrian Arab Republic” OR Tajikistan OR Thailand OR Timor‐Leste OR Timor OR Turkey OR Turkmenistan OR Uzbekistan OR Vietnam OR “West Bank” OR Gaza OR Yemen OR Comoros OR Maldives OR Mauritius OR Seychelles).mp
− (“Pacific Islands” OR “American Samoa” OR Fiji OR Guam OR Kiribati OR “Marshall Islands” OR Micronesia OR New Caledonia OR “Northern Mariana Islands” OR Palau OR “Papua New Guinea” OR Samoa OR “Solomon Islands” OR Tonga OR Tuvalu OR Vanuatu).mp
*Systematic review key words*
− ((systematic* or synthes*) adj3 (research or evaluation* or finding* or thematic* or report or descriptive or explanatory or narrative or meta* or review* or data or literature or studies or evidence or map or quantitative or study or studies or paper or impact or impacts or effect* or compar*)).ti,ab,sh
OR
(“meta regression” or “meta synth*” or “meta‐synth*” or “meta analy*” or “metaanaly*” or “meta‐analy*” or “metanaly*” or “metaregression” or “metaregression” or “methodologic* overview” or “pool* analys*” or “pool* data” or “quantitative* overview” or “research integration”).ti,ab,sh
OR
(review adj3 (effectiveness or effects or systemat* or synth* or integrat* or map* or methodologic* or quantitative or evidence or literature)).ti,ab,sh
*Qualitative review search term*
(((“meta ethnography” OR “meta ethnographic”) OR (“meta synthesis”) OR (synthesis AND (“qualitative literature” OR “qualitative research”)) OR (“critical interpretive synthesis”) OR (“systematic review” AND (“qualitative research” OR “qualitative literature” OR “qualitative studies”)) OR (“thematic synthesis” OR “framework synthesis”) OR (“realist review” OR “realist synthesis”) OR (((“qualitative systematic review” OR “qualitative evidence synthesis”)) OR (“qualitative systematic reviews” OR “qualitative evidence syntheses”)) OR ((“quality assessment” OR “critical appraisal”) AND (“qualitative research” OR “qualitative literature” OR “qualitative studies”)) OR ((“literature search” OR “literature searching” OR “literature searches”) AND (“qualitative research” OR “qualitative literature” OR “qualitative studies”)) OR (Noblit AND Hare)) OR (“meta narrative” OR “meta narratives” OR “narrative synthesis”)
*Disability key words*
− ((Disable* or Disabilit* or Handicapped) adj5 (person* or people or child*or adolescen* or women or mother*or maternal, group)).sh,ti,ab
− ((physical* or intellectual* or learning or psychiatric* or sensory or motor or neuromotor or cognitive or mental* or developmental or communication or learning) adj2 (disabilit* or disabl* or handicap*)).ti,ab
− ((cognitive* or learning or mobility or sensory or visual* or vision or sight or hearing or physical* or mental* or intellectual*) adj2 impair*).ti,ab
− ((mental health or mental disorder* or depress* or anxiety or psychiat* or well‐being or quality of life or self‐esteem or self perception)).ti,ab
− ((mental* or emotional* or psychiatric or neurological or neurologic) adj2 (disorder* or ill or illness*)).ti,ab (deaf or deafness or blind or blindness).ti,ab
− exp Disabled persons/
− (Autis* or Dyslexi* or Down* Syndrome or Mongolism or Trisomy 21).sh,ti,ab
− exp Intellectual disability/or exp Developmental Disabilities/ or exp Child Development Disorders, Pervasive/ or exp Communication Disorders/
− ((Intellectual* or Educational*or Mental* or Psychological* or Developmental) adj5 (impair* or retard* or deficienc* or Deficien* or disable* or disabili* or handicap* or ill*)).sh,ti,ab
− ((Hearing or Acoustic or Ear*) adj5 (loss* or impair* or deficienc* or disable* or disabili* or handicap*)).sh,ti,ab
− ((Visual* or Vision or Eye*) adj5 (loss* or impair* or deficienc* or disable* or disabili* or handicap*)).sh,ti,ab
− (Deaf* or Blind*).sh,ti,ab
− exp Cerebral palsy/or exp Spina Bifida Cystica/or exp Spina Bifida Occulta/or exp Muscular dystrophies/or exp Arthritis/ or exp Osteogenesis Imperfecta/or exp Musculoskeletal Abnormalities/or exp Brain Injuries/ or exp Amputation/or exp Clubfoot/or exp Poliomyelitis/or exp Paraplegia/or exp Hemiplegia/or exp Stroke
− (Cerebral pals* or Spina bifida or Muscular dystroph* or Arthriti* or Osteogenesis imperfecta or Musculoskeletal abnormalit* or Musculo‐skeletal abnormalit* or Muscular abnormalit* or Skeletal abnormalit* or Limb abnormalit* or Brain injur* or Amputation* or Clubfoot or Poliomyeliti* or Paraplegi* or Paralys* or Paralyz* or Hemiplegi* or Stroke* or Cerebrovascular accident*).sh,ti,ab
− (Physical* adj5 (impair* or deficienc* or disable* or disabili* or handicap*)).sh,ti,ab

## APPENDIX D Coding tool

1. Study design
  - Systematic reviews [Selectable]
  - RCT [Selectable]
  - Quasi‐experimental study [Selectable]
  - Case‐control [Selectable]
  - Cohort [Selectable]
  - Controlled trial [Selectable]
  - Publication status
  - Completed [Selectable]
  - On‐going [Selectable]
2. Population
  - People with disabilities [Selectable]
  - Children [Selectable]
  - Women [Selectable]
  - Conflict affected [Selectable]
  - Elderly [Selectable]
  - Disadvantaged [Selectable]
  - Migrants [Selectable]
  - Ethnic minorities [Selectable]
  - Adults [Selectable]
3. Region
  - South Asia [Selectable]
  - Sub‐Saharan Africa [Selectable]
  - East Asia and Pacific [Selectable]
  - Europe and Central Asia [Selectable]
  - Latin America and Caribbean [Selectable]
  - Middle East and North Africa [Selectable]
  - North America [Selectable]
4. Low income countries [Selectable]
  - Afghanistan [Selectable]
  - Rwanda [Selectable]
  - Somalia [Selectable]
  - South Sudan [Selectable]
  - Zimbabwe [Selectable]
  - Uganda [Selectable]
  - Nepal [Selectable]
  - Niger [Selectable]
  - Ethiopia [Selectable]
  - Eritrea [Selectable]
  - Liberia [Selectable]
  - Congo [Selectable]
  - Burundi [Selectable]
5. Lower‐middle‐income countries [Selectable]
  - Armenia [Selectable]
  - Indonesia [Selectable]
  - Philippines [Selectable]
  - Sri Lanka [Selectable]
  - Kenya [Selectable]
  - Bangladesh [Selectable]
  - Cambodia [Selectable]
  - Lesotho [Selectable]
  - Egypt, Arab Rep [Selectable]
  - India [Selectable]
  - Pakistan [Selectable]
  - Nigeria [Selectable]
  - Vietnam [Selectable]
  - Zambia [Selectable]
  - Ghana [Selectable]
  - Bolivia [Selectable]
  - Ukraine [Selectable]
6. Upper‐middle‐income countries [Selectable]
  - Iraq [Selectable]
  - Romania [Selectable]
  - Turkey [Selectable]
  - Iran [Selectable]
  - China [Selectable]
  - Lebanon [Selectable]
  - Brazil [Selectable]
  - South Africa [Selectable]
  - Thailand [Selectable]
  - Russia [Selectable]
  - Peru [Selectable]
  - Jamaica [Selectable]
  - Malaysia [Selectable]
  - Argentina [Selectable]
  - Libya [Selectable]
7. High income countries [Selectable]
8. High fragility (FCAS)
  - Somalia [Selectable]
  - Afghanistan [Selectable]
  - South Sudan [Selectable]
  - Eritrea [Selectable]
  - Syria [Selectable]
  - Chad [Selectable]
  - Libya [Selectable]
  - Venezuela [Selectable]
  - CAR [Selectable]
  - Pakistan [Selectable]
  - Yemen [Selectable]
  - Ukraine [Selectable]
  - Sudan [Selectable]
  - Burundi [Selectable]
  - North Korea [Selectable]
  - Myanmar [Selectable]
  - DRC [Selectable]
  - Nigeria [Selectable]
  - Iraq [Selectable]
9. Moderate fragility (FCAS)
  - Zimbabwe [Selectable]
  - Tajikistan [Selectable]
  - Lebanon [Selectable]
  - Guinea [Selectable]
  - Congo, Rep [Selectable]
  - Azerbaijan [Selectable]
  - Haiti [Selectable]
  - Mauritania [Selectable]
  - Turkmenistan [Selectable]
  - Cameroon [Selectable]
  - Iran [Selectable]
  - Uzbekistan [Selectable]
  - Egypt [Selectable]
  - Guinea‐Bissau [Selectable]
10. Low fragility (FCAS)
  - Kyrgyz republic [Selectable]
  - Liberia [Selectable]
  - Djibouti [Selectable]
  - Angola [Selectable]
  - Ethiopia [Selectable]
  - Mali [Selectable]
  - Bangladesh [Selectable]
  - Gambia [Selectable]
  - OPTs [Selectable]
  - Kenya [Selectable]
  - Madagascar [Selectable]
  - Nepal [Selectable]
  - Comoros [Selectable]
  - Niger [Selectable]
  - Algeria [Selectable]
  - Honduras [Selectable]
11. Neighbours (FCAS)
  - Benin [Selectable]
  - Zambia [Selectable]
  - Tanzania [Selectable]
  - Uganda [Selectable]
  - Rwanda [Selectable]
  - Jordan [Selectable]
  - Thailand [Selectable]
  - Laos [Selectable]
12. Fragile and conflict‐affected situation (FCAS) [Selectable]
13. Countries
14. Interventions

- Health [Selectable]
  - Promotion [Selectable]
  - Prevention [Selectable]
  - Medical care [Selectable]
  - Rehabilitation [Selectable]
  - Assistive devices [Selectable]

- Education [Selectable]
  - Early child development [Selectable]
  - Nonformal [Selectable]
  - Primary and secondary [Selectable]
  - Lifelong learning [Selectable]

- Livelihood [Selectable]
  - Skills development [Selectable]
  - Self‐employment [Selectable]
  - Waged employment [Selectable]
  - Financial services [Selectable]

- Social protection [Selectable]
  - Social [Selectable]
  - Relationship, marriage and family [Selectable]
  - Personal assistance [Selectable]
  - Culture, religion and arts [Selectable]
  - Sports, recreation and leisure [Selectable]
  - Access to justice [Selectable]

- Empowerment [Selectable]
  - Social mobilisation [Selectable]
  - Political Participation [Selectable]
  - Language and communication [Selectable]
  - Self‐help groups & Disabled People's Organisation [Selectable]

- Advocacy and Governance
  - Advocacy and Governance [Selectable]

15. Outcomes

- Health [Selectable]
  - Mental health and cognitive development [Selectable]
  - Access to health services [Selectable]
  - Immunisation [Selectable]
  - Health check‐up [Selectable]
  - Rehabilitation [Selectable]
  - Access to assistive devices [Selectable]
  - Nutrition [Selectable]
  - Morbidity and mortality [Selectable]

- Education [Selectable]
  - Enrolment to primary, secondary and tertiary education [Selectable]
  - Attendance [Selectable]
  - Education in mainstream education facilities/inclusive education [Selectable]
  - Social and life skill development [Selectable]
  - Access to educational services [Selectable]

- Livelihood [Selectable]
  - Employment in formal and informal sector [Selectable]
  - Access to job market [Selectable]
  - Control over own money [Selectable]
  - Access to financial services such as grants and loans [Selectable]
  - Poverty and out‐of‐pocket payment [Selectable]
  - Access to social protection programmes [Selectable]
  - Participation in development of inclusive policies [Selectable]

- Social [Selectable]
  - Stigma and discrimination [Selectable]
  - Safety [Selectable]
  - Participation in mainstream recreational, leisure and sports activity [Selectable]
  - legal rights [Selectable]
  - Access to justice [Selectable]
  - Participation in cultural and religious activity [Selectable]
  - Interpersonal interaction and relationships [Selectable]
  - Social identity and responsibilities [Selectable]

- Empowerment
  - Informed choices [Selectable]
  - Positions in public institutions and Judiciary [Selectable]
  - Voting rights [Selectable]
  - Representation at community level [Selectable]
  - Advocacy [Selectable]

16. Systematic review quality
  - Low [Selectable]
  - Moderate [Selectable]
  - High [Selectable]
  - Impact evaluation [Selectable]
  - Protocol [Selectable]
17. Type of impairment
  - Physical impairment [Selectable]
  - Visual impairment [Selectable]
  - Mental impairment [Selectable]
  - Hearing impairment [Selectable]

Intellectual/learning impairment [Selectable]

## References

[cl21006-bib-0001] Asian Development Bank (2005). Disability brief: Identifyingand addressing the needs of disabled people. Banque Asiatique du Développement. Mandaluyong, Philippines. Retrieved fromhttp://www2.adb.org/Documents/Reports/Disabled‐People‐Development/disability‐brief.pdf

[cl21006-bib-0002] Andresen, E. M , Lollar, J. L. , & Meyers, R. M. (2000). Disability outcomes research: Why this supplement, on this topic, at this time? Archives of Physical Medicine and Rehabilitation, 81, S1–S4. 10.1053/apmr.2000.20614 11128898

[cl21006-bib-0003] Banks, L. M. , Kuper, H. , & Polack, S. (2017). Poverty and disability in low‐ and middle‐income countries: A systematic review. PLoS One, 12(12), e0189996. 29267388 10.1371/journal.pone.0189996 PMC5739437

[cl21006-bib-0004] CBM (2012). A human rights‐based approach to disability in development. Retrieved from http://www.cbm.org/A‐human‐rights‐based‐approach‐to‐disability‐in‐development‐396302.php

[cl21006-bib-0005] CRC (1989). Convention on the rights of the child. Retrieved from http://www.ohchr.org/EN/ProfessionalInterest/Pages/CRC.aspx

[cl21006-bib-0006] DEID (2000). Disability, Poverty and Development. London, UK: DFID.

[cl21006-bib-0007] Devon, R. , Lydon, S. , Healy, O. , & McCoy, A. (2016). A systematic review of the effectiveness of precision teaching for individuals with developmental disabilities. Review Journal of Autism and Developmental Disorders, 3(3), 179–195.

[cl21006-bib-0008] Glickman, S. , Anstrom, K. , Lin, L. , Chandra, A. , Laskowitz, D. , Woods, C. , … K. , M. (2008). Challenges in enrollment of minority, pediatric, and geriatric patients in emergency and acute care clinical research. Annals of Emergency Medicine, 51(6), 775–780. 18191297 10.1016/j.annemergmed.2007.11.002

[cl21006-bib-0009] Helander (1989). Training in the community for people with disabilities. Geneva: World Health Organization.

[cl21006-bib-0010] Iemmi, V. , Gibson, L. , Blanchet, K. , Kumar, S. , Rath, S. , Hartley, S. , … Kuper, H. (2015). Community‐based rehabilitation for people with disabilities in low‐and middle‐income countries: A systematic review. Campbell Systematic Reviews

[cl21006-bib-0011] ILO/UNESCO/WHO . (2004). A strategy for rehabilitation, equalization of opportunities, poverty reduction and social inclusion of people with disabilities. International Labour Organization, United Nations Educational, Scientific and Cultural Organization, and World Health Organization.

[cl21006-bib-0012] Metts, R. , & Mondiale, B. (2004). Disability and development, background paper for the World Bank. World Bank. Washington D.C. Retrieved from http://siteresources.worldbank.org/DISABILITY/Resources/280658‐1172606907476/mettsBGpaper.pdf

[cl21006-bib-0013] Mondiale, B. (2007). Social analysis and disability: A guidance note. World Bank. New York. Retrieved from http://siteresources.worldbank.org/EXTSOCIALDEV/Resources/3177394‐1175102311639/3615048‐1175607868848/SocialAnalysis&Disability‐Full.pdf

[cl21006-bib-0014] Shea, B. J. , Grimshaw, J. M. , Wells, G. A. , Boers, M. , Andersson, N. , Hamel, C. , … Bouter, L. M. (2007). Development of AMSTAR: A measurement tool to assess the methodological quality of systematic reviews. BMC Medical Research Methodology, 7, 10. 17302989 10.1186/1471-2288-7-10 PMC1810543

[cl21006-bib-0015] UNCRPD (2006). The United Nation Convention on the Rights of Person with Disabilities. Retrieved from https://www.un.org/development/desa/disabilities/convention‐on‐the‐rights‐of‐persons‐with‐disabilities/convention‐on‐the‐rights‐of‐persons‐with‐disabilities‐2.html

[cl21006-bib-0016] United Nations—Disability Department of Economic and Social Affairs . (n.d.). Sustainable development goals (SDGs) and disability. Retrieved from https://www.un.org/development/desa/disabilities/about‐us/sustainable‐development‐goals‐sdgs‐and‐disability.html

[cl21006-bib-0017] United Nations Development Programme (2010). Inclusive development. United Nations Development Programme.

[cl21006-bib-0018] USAID (1997). USAID disability policy paper. US Agency for International Development, Washington D.C.

[cl21006-bib-0019] USAID (1997). USAID disability. U.S. Agency for International Development, Washington D.C.

[cl21006-bib-0020] WHO (2011). World report on disability. Geneva: World Health Organisation. Retrieved from. http://www.who.int/disabilities/world_report/2011/report.pdf.

[cl21006-bib-0021] World Health Organisation (2010). World Health Statistics 2010.

[cl21006-bib-0022] World Health Organisation (2014). World Health Statistics 2014.

[cl21006-bib-0023] World Health Organisation (2013). World Health Statistics 2013.

[cl21006-bib-0024] World Health Organization (2001). International classification of functioning, disability and health: ICF, World Health Organisation, Geneva

[cl21006-bib-0025] World Bank Classifications (2016). Retrieved from https://datahelpdesk.worldbank.org/knowledgebase/articles/378834‐how‐does‐the‐world‐bank‐classify‐countries

[cl21006-bib-0026] WPA (1982). World Programme of Action Concerning Disabled Persons. Retrieved from https://www.un.org/development/desa/disabilities/resources/world‐programme‐of‐action‐concerning‐disabled‐persons.html

[cl21006-bib-0027] Üstün, T. , Murray, C. , & Evans, D. (2003). The World Health Surveys: Health systems performance assessment: Debates, methods and empiricism, World Health Organisation, Geneva.

